# Spindle Cell Carcinoma of Buccal Mucosa: An Unusual Presentation of Squamous Cell Carcinoma

**DOI:** 10.7759/cureus.57007

**Published:** 2024-03-26

**Authors:** Husna Tehzeeb, Alka Hande, Aayushi Pakhale, Ankita Chavhan, Sakshi Akolkar

**Affiliations:** 1 Oral Pathology and Microbiology, Sharad Pawar Dental College and Hospital, Datta Meghe Institute of Higher Education and Research, Wardha, IND

**Keywords:** histopathology, oral carcinoma, biphasic tumor, oral squamous cell carcinoma, spindle cell carcinoma

## Abstract

Oral squamous cell carcinoma (OSCC) is the most common carcinoma in the H&N (head and neck) region, in which squamous cells show variability in differentiation like basaloid, glandular, and spindle cells. Spindle cell carcinoma (SpCC) is an unusual variant of SCC that is aggressive in nature and has the ability to recur and metastasize. The presence of malignant mesenchymal and squamous epithelial cells gives it a biphasic nature. So, we present a case of SpCC of buccal mucosa in a 45-year-old male who had an ulcerated growth on his left buccal mucosa that had been present for two years.

## Introduction

Spindle cell carcinoma (SpCC), also known as sarcomatoid squamous cell carcinoma, is a rare biphasic form of squamous cell carcinoma (SCC) that consists of both epithelial and mesenchymal neoplastic constituent [1]. This disease entity is classified as malignant epithelial tumors of squamous cell origin in the classification system given by WHO of oral cavity and oropharynx tumors and is designated as SpCC. Histopathologically, SpCC may also be graded moderately and poorly differentiated squamous cell carcinoma (SCC) which comprises elongated and spindled shaped epithelial cells [2]. The interface of the two components may be gradual or abrupt. SpCC is more frequently seen in males and usually appears between 50 and 70 years old individuals but is very rare in childhood and adolescence. SpCC typically occurs in the pharynx and oral cavity but less frequently in the sinonasal area and pharynx. The risk factors include alcohol use, smoking, poor oral hygiene, and people with a history of irradiation to the head and neck area [3]. SpCC exhibits variability in clinical characteristics and behaviour. The common presentation includes ulceration, polypoid, fungating, or exophytic growth, which has a significant impact on its management. There is a dearth of reported cases in the literature. Therefore, here we present a case study detailing a rare occurrence of SpCC affecting the buccal mucosa of a man aged 45 years who sought treatment at our institution due to a chief complaint of an ulcerated growth on the facial region to the left side.

## Case presentation

**Figure 1:**
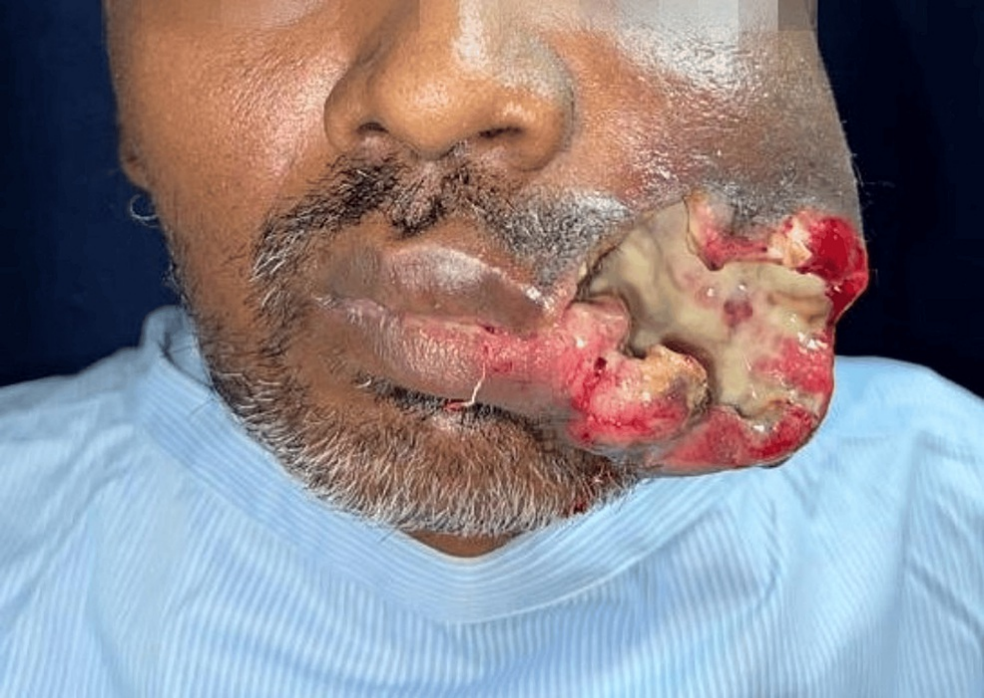
Clinical presentation of an ulceroproliferative lesion over the left gingivobuccal sulcus of the patient

A man aged 45 years visited the outpatient department (OPD) of our institute with a painless unhealing ulcer over the left side of his face in the cheek region for two years approximately. The patient didn't provide noteworthy details about their previous medical or dental conditions, but he did mention a history of chewing tobacco for the past 12 years. Clinically, During the extraoral examination, facial asymmetry was observed on the face due to an extraoral fungating lesion present on the left side, which extended from the corner of the mouth to the angle of the mandible in an anteroposterior direction, and superior-inferiorly from infraorbital region to inferior border of mandible on left side measuring 10 x 8 cm approximately. The lesion was roughly oval in shape, whitish in colour, with everted margins and irregular edges. On palpation tenderness was present. Induration was present and consistency ranged from soft to firm. Temporomandibular joint movements were restricted with reduced mouth opening and incompetent lips (Figure 1).

**Figure 2:**
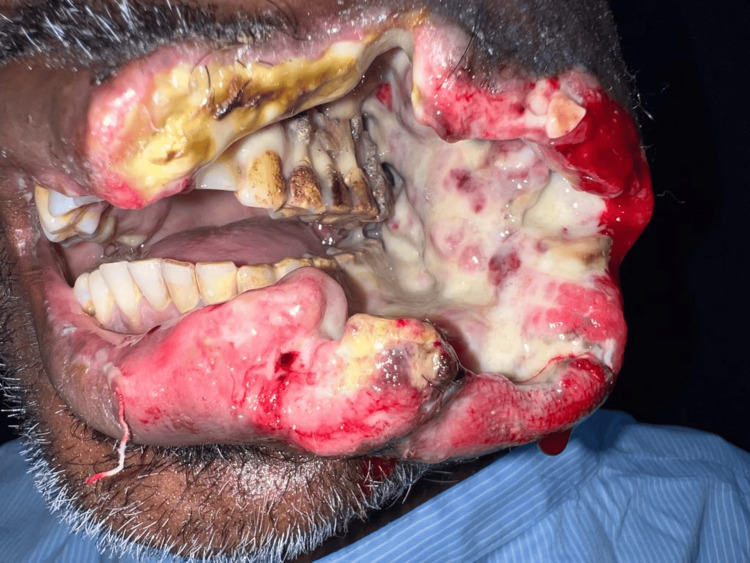
Intraoral lesion extending from the corner of the mouth to RMT on left side RMT: Retromolar trigone.

During the intraoral examination, a solitary, ulcerative, and proliferative lesion was observed on the left buccal mucosa. This lesion extended in an anteroposterior direction from the left corner of the mouth to the distal aspect of tooth 48, adjacent to the retromolar trigone (RMT) region. Additionally, it extended in a superoinferior direction from the depth of the upper buccal vestibule to the depth of the lower buccal vestibule, measuring approximately 10 × 8 cm. The lesion appeared firm and non-tender with indistinct borders (Figure 2).

**Figure 3:**
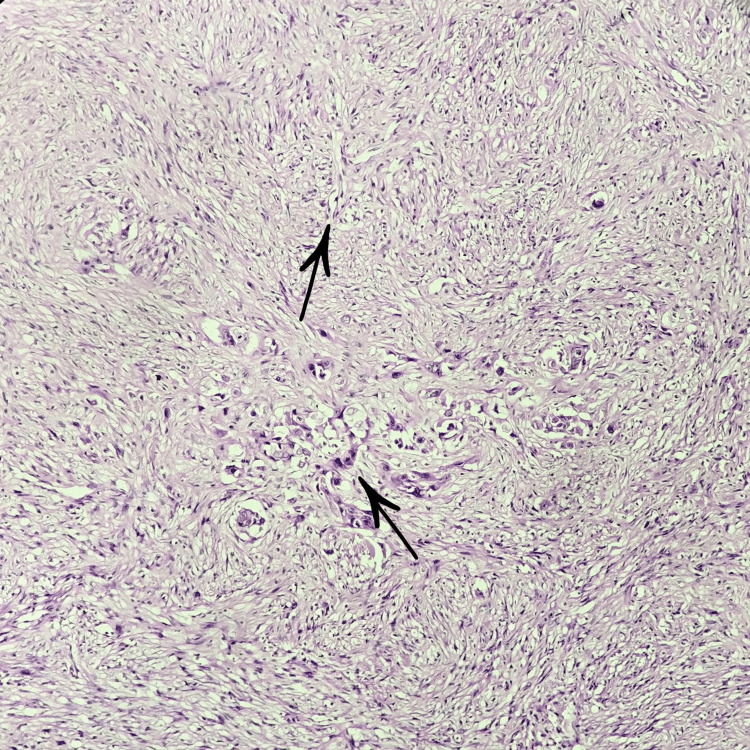
Histopathological examination under low-power microscopy (4X) Biphasic tumor showing epithelial and mesenchymal components.

The tissue was subjected to incisional biopsy, and on histopathological examination under low power microscopic view, two patterns of tumor cells were seen suggestive of a biphasic tumor. There were spindle shaped cells dispersed in fibrocellular stroma in streaming pattern, along with them keratinising dysplastic squamous cells were also evident (Figure 3).

**Figure 4:**
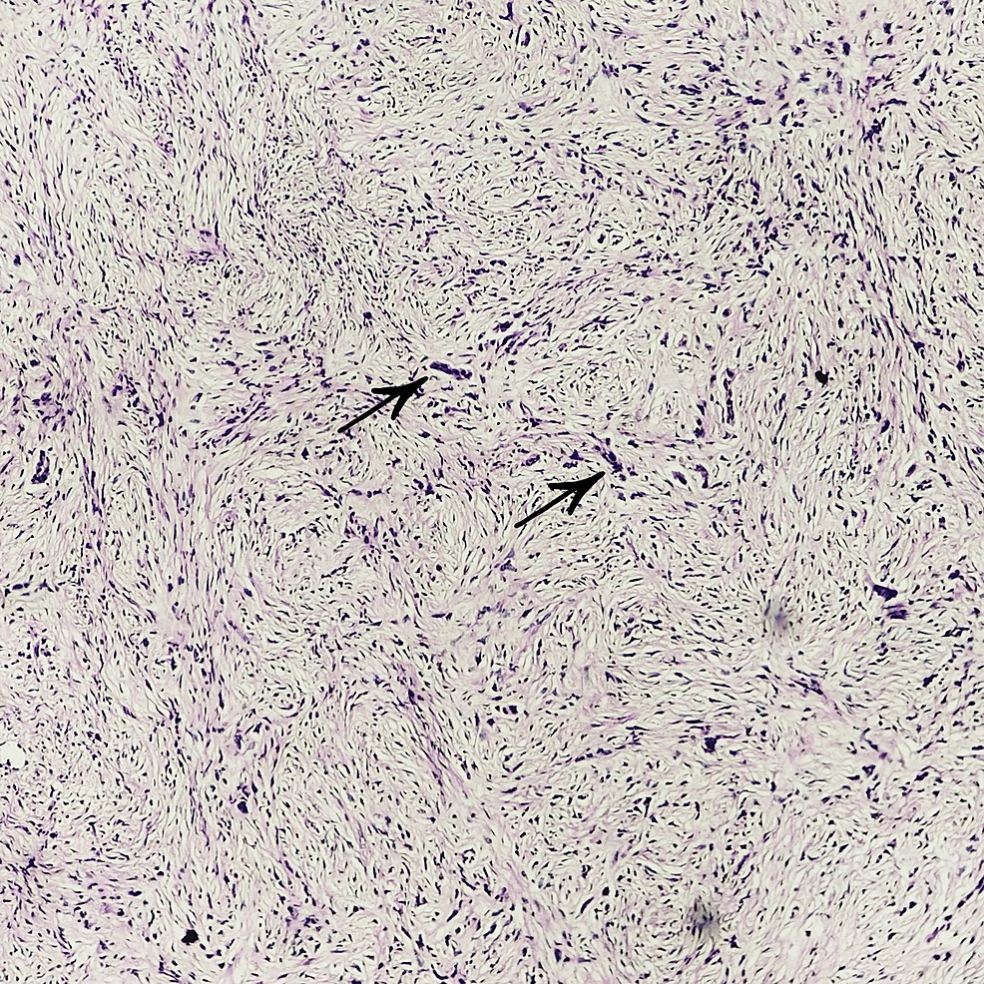
Histopathological examination image I under high-power microscopy (40X) Elongated spindled-shaped and fusiform giant cells are present within fibro cellular stroma.

**Figure 5:**
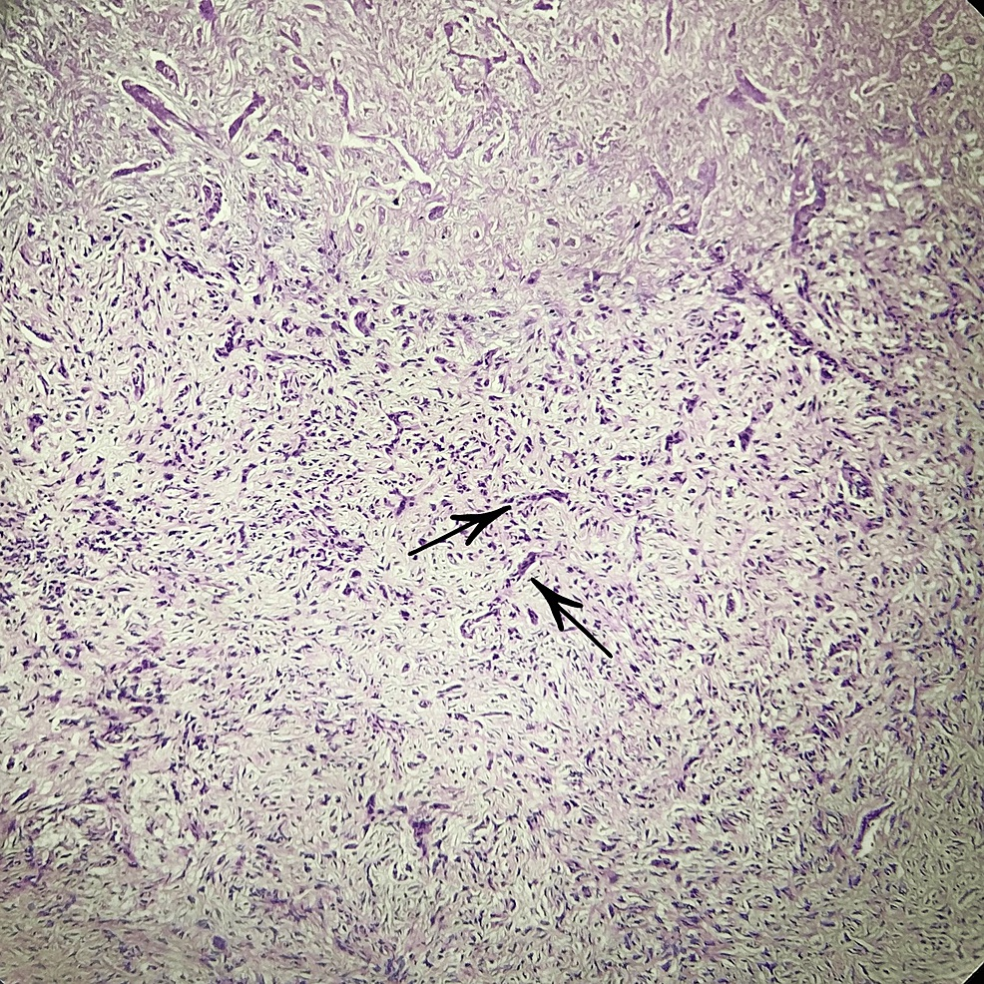
Histopathological examination image II under high-power microscopy (40X) Syncytium aggregates are present within fibro cellular stroma.

Under high power view, malignant epithelial cells were elongated, spindle-shaped, having large, hyperchromatic, pleomorphic, vesiculated nuclei with bizarre mitotic figures and increased nuclear/cytoplasmic (N/C) ratio. In places, the presence of giant cells was also seen (Figure 4). Syncytium aggregates were also seen in some areas of the fibro cellular stroma (Figure 5).

**Figure 6:**
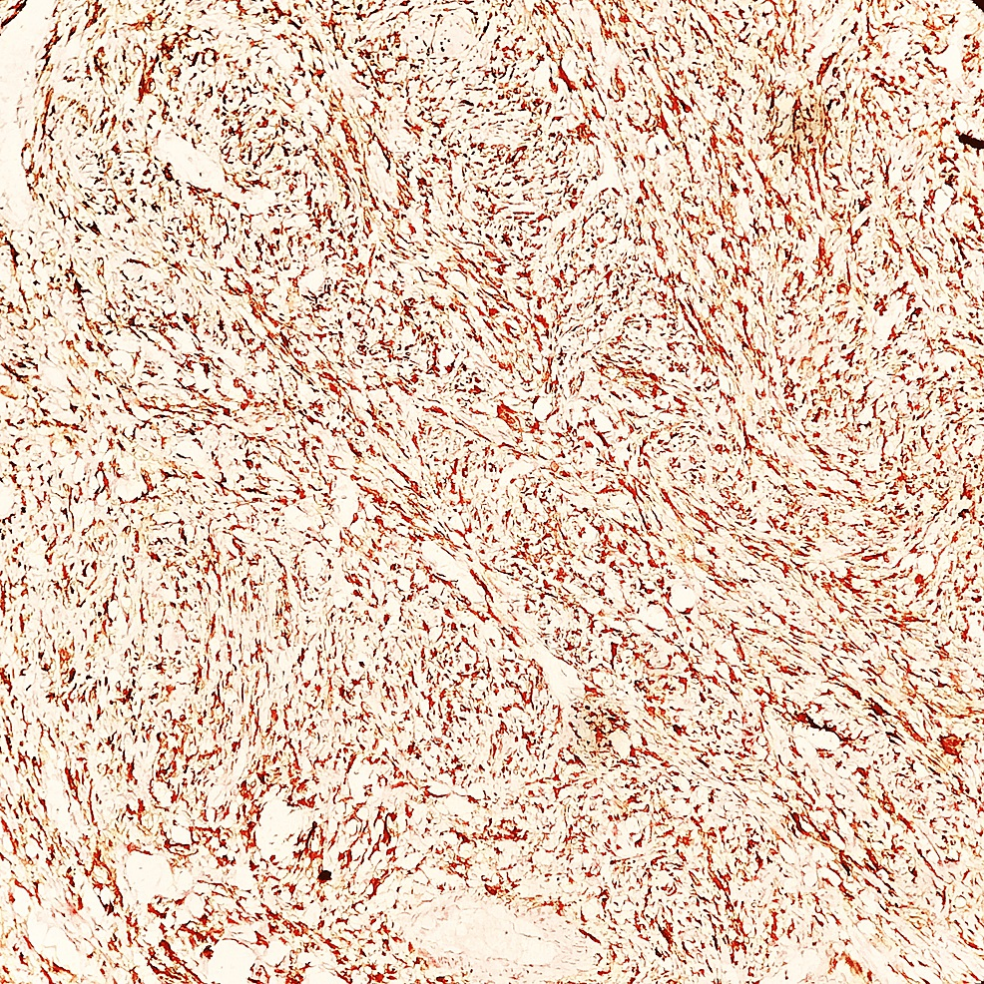
Immunohistochemical analysis image An immunohistochemical study image showing spindle cells strongly positive for cytokeratin (10X).

Upon immunohistochemical analysis, the spindle cell component exhibited strong cytokeratin positivity, thus confirming the epithelial nature of the neoplastic cells (Figure 6). Following the surgical removal of the lesion by wide local excision from the corner of the mouth to RMT, hemi-maxillary and hemi-mandibulectomy along with bilateral radical neck dissection (type III) performed and reconstructed with bipaddled pectoralis major myocutaneous (PMMC) flap under general anaesthesia, the patient underwent histopathological examination which revealed clear margins in the excised specimen, yet indicated lymphovascular and perineural invasion. A total of 33 lymph nodes were identified, out of which six were positive for invasion and the carcinoma was graded as poorly differentiated. At the six-month follow-up, the patient reported a recurrence of the condition, prompting another surgical intervention. Subsequent to 12 months of follow-up, there was no detectable evidence of the disease.

## Discussion

SpCC, a rare and unusual variant of SCC, consists of malignant spindle and squamous cell types derived from epithelial tissue. The mesenchymal appearance in this tumor can be attributed to the spindle cell component. Additionally, diagnosing SpCC can be difficult, particularly when its squamous component is not obvious [4]. SpCC represents less than 1% of all carcinomas in the head and neck (H&N) region [5]. It predominantly impacts males in their sixth and seventh decades of life [4]. as seen in our case. The laryngeal mucosa is the most commonly affected site by SpCC, but occasionally it can also be seen in the upper aerodigestive tract, which includes the nasal cavity and hypopharynx, oesophagus and, in the oral cavity gingiva, buccal mucosa and tongue. In this case, buccal mucosa was involved which is not a common site for SpCC. It also shows a very rare predilection for skin and breasts also [6]. The spindle cell component can mimic a wide range of lesions, which can be reactive, benign lesions like granulation tissue induced by radiations, or malignant lesions like fibrosarcoma. Therefore, any indication of an epithelial element should be carefully sought in suspected lesions so as to establish a correct diagnosis [4].

Clinically, the characteristic growth pattern of SpCC denotes its distinctive clinic-pathologic characteristic. More than 90% of tumors of the larynx and pharynx appear as “polypoid and exophytic masses” that protrude in the cavity [7,8]. The growth pattern in the oral cavity is variable, with fifty to sixty percent of tumors being exophytic, whereas others are present with ulcerative lesions [9], this case illustrated an ulcerative lesion, rendering it even more uncommon.

On histopathological aspects, the specific pathogenesis of this spindle cell differentiation was a subject of debate for many years, giving rise to several alternative terms, such as “metaplastic carcinoma, pleomorphic carcinoma, carcinosarcoma, and pseudosarcoma” [10]. Many studies examining the “morphologic, immunohistochemical, ultrastructural, and molecular features” of SpCC have demonstrated over time that the spindle cell constituent represents divergent differentiation of squamous cells. The spindle cell population of the tumor has revealed epithelial characteristics proven by numerous genetic studies [11-13]. The histogenetic nature of spindle cells has been explained by three distinct theories. The first theory suggests that different stem cells give rise to epithelial and spindle cells at the same time, which is how the name "collision" tumour was coined. According to the second idea, the "spindle component" of the tumor is suggested as a stromal atypical reactive growth, leading to its classification as a "pseudosarcoma". Lastly, it states that spindle cells have undergone "dedifferentiation" or "transformation" into epithelial cells and that both types of cells have the same monoclonal origin [4]. The squamous component may manifest as either "localized dysplasia, carcinoma in situ, or clearly invasive squamous cell carcinoma”. The latter characteristic is commonly observed in the stalk of the polyp, either extending in the advancing front of the tumor or at its deepest point. Spindle cells frequently demonstrate a "falling off" effect from the surface while the dysplastic squamous epithelium remains present in its basal layer [7,9]. The spindle cells can exhibit either bland and uniform characteristics or they may display significant variability with multinucleated giant cells within the tumor. Various architectural patterns can be observed, including "fascicular," "storiform," lace-like, or "myxoid." Occasionally, distinct sarcomatous differentiation may be present, such as "osteosarcomatous," "chondrosarcomatous," or "rhabdomyosarcomatous" [14].

The line of treatment for SpCC and squamous cell carcinomas of the same clinical stage is similar. The best choice of treatment for treating tumors in oral region is surgery, sometimes with neck dissection, followed by chemotherapy or radiotherapy [15,16]. The use of radiotherapy, particularly for oral malignancies, is also debatable and role of chemotherapeutics in treatment of SpCC is still not clear [17,18].

## Conclusions

We describe an unusual variant of oral squamous cell carcinoma distinguished by spindle cell differentiation, specifically SpCC of the buccal mucosa. In view of the dearth of cases in the literature, limited knowledge is present about its precise aetiology, clinical manifestations, and long-term prognosis. The application of radiotherapeutic techniques is increasing nowadays because of the rise in conservative treatment approaches to the disease. Though this tumor has a challenging character, the histopathological diagnosis was guided by the patient's history, the presence of squamous cell constituent, and the identification of the spindle cells of epithelial origin. Sarcomas in the head and neck area are very rare; therefore, when assessing an ulceroproliferative lesion in these regions containing spindle cells, one should always consider the possibility of spindle cell carcinomas.
